# Substantial variability in what is considered important in the radiological report for anterior shoulder instability: a Delphi study with Dutch musculoskeletal radiologists and orthopedic surgeons

**DOI:** 10.1016/j.jseint.2024.03.012

**Published:** 2024-04-08

**Authors:** Cain Rutgers, Lukas P.E. Verweij, Michel P.J. van den Bekerom, Henk-Jan van der Woude, SINC Study Group, A.E. Scholtens, A. Soepboer, A. van Noort, B. Muller, B.E. Steunenberg, C.P.J. Visser, D.V. Loeffen, D.F. Hanff, D.F.P. van Deurzen, E.E.J. Raven, E.H.G. Oei, F.M. Zijta, H.C. van der Veen, I.D. Kilsdonk, J. Dening, J. Habets, L. Kluijtmans, L.E. Huygen, M.F. Boomsma, M.E.A.P.M. Adriaensen, J.O. van der Meer, F.O. Lambers Heerspink, O.A.J. van der Meijden, P.H. Ousema, R.G. Krol, S.M. Bollen, S.C.E. Diepstraten, S.N. de Jong, T.D. Berendes, T. Gosens, T.D.W. Alta, V.E. Versteegh, W. Foppen, Y.V. Kleinlugtenbelt

**Affiliations:** a Faculty of Behavioural and Movement Sciences, Department of Human Movement Sciences, Vrije Universiteit Amsterdam, Amsterdam, the Netherlands; b Amsterdam Movement Sciences, Musculoskeletal Health Program, Amsterdam, the Netherlands; c Shoulder and Elbow Unit, Joint Research, Department of Orthopedic Surgery, OLVG, Amsterdam, the Netherlands; d Amsterdam Shoulder and Elbow Center of Expertise (ASECE), Amsterdam, the Netherlands; e Amsterdam UMC, Location AMC, Department of Orthopaedic Surgery and Sports Medicine, University of Amsterdam, Amsterdam, the Netherlands; f Department of Orthopaedic Surgery, Medical Center Jan van Goyen, Amsterdam, the Netherlands; g Shoulder and Elbow Unit, Joint Research, Department of Radiology, OLVG, Amsterdam, the Netherlands

**Keywords:** Shoulder, Shoulder dislocation, X-rays, Magnetic resonance imaging, Tomography, X-ray computed, Bankart lesions, Delphi technique

## Abstract

**Background:**

Standardized consensus-based radiological reports for shoulder instability may improve clinical quality, reduce heterogeneity, and reduce workload. Therefore, the aim of this study was to determine important elements for the x-ray, magnetic resonance imaging (MRI) arthrography (MRA), and computed tomography (CT) report, the extent of variability, and important MRI views and settings.

**Methods:**

An expert panel of musculoskeletal radiologists and orthopedic surgeons was recruited in a three-round Delphi design. Important elements were identified for the x-ray, MRA, and CT report and important MRI views and setting. These were rated on a 0-9 Likert scale. High variability was defined as at least one score between 1-3 and 7-9. Consensus was reached when ≥80% scored an element 1-3 or 7-9.

**Results:**

The expert panel consisted of 21 musculoskeletal radiologists and 15 orthopedic surgeons. The number of elements identified in the first round was seventeen for the x-ray report, 52 for MRA, 21 for CT, and 23 for the MRI protocol. The number of elements that reached consensus was five for x-ray, twenty for MRA, nine for CT, and two for the MRI protocol. High variability was observed in 76.5% (n = 13) x-ray elements, 85.0% (n = 45) MRA, 76.2% (n = 16) CT, and 85.7% (n = 18) MRI protocol.

**Conclusion:**

Substantial variability was observed in the scoring of important elements in the radiological for the evaluation of anterior shoulder instability, regardless of modality. Consensus was reached for five elements in the x-ray report, twenty in the MRA report, and nine in the CT report. Finally, consensus was reached on two elements regarding MRA views and settings.

Shoulder instability research is characterized by heterogeneous outcomes and patient groups.^23^^,^^28^^,^^14^^,^^29^ In order to reduce the heterogeneity for future research, several consensus studies have been performed to determine a standardized outline for diagnosis, management, surgical report, and set of outcomes.^11^^,^^12^^,^^18^^,^^25^^,^^13^^,^^31^^,^^30^ For example, consensus was reached on a set of risk factors for recurrence following nonoperative or surgical management, including gender, age, mechanism of injury, sports, hyperlaxity, bone loss, arthritic changes, and prior surgeries.^11^^,^^12^ However, a consensus-based standardized radiological report is lacking in the current literature. Despite the presence of lesions playing a key role in determining the risk of recurrence, and in most optimal treatment and most shoulder instability research, there is no consensus on how shoulder lesions are reported and defined.^1^^,^^22^^,^^33^ This leads to substantial heterogenic data between and within studies or missing data, resulting in conflicting results.^23^^,^^35^

A consensus-based standardized radiological report for the various imaging techniques may support in improving research quality, sharing data, and reducing bias.^20^^,^^30^ In the evaluation of shoulder instability, the various imaging techniques include x-ray, magnetic resonance imaging (MRI), and computed tomography (CT) techniques. MRI arthrography (MRA) and CT arthrography (CTA) make use of intra-articular contrast to increase visibility of intra-articular structures including the labro-ligamentary complex, MRA most widely used.^34^ Depending on whether acute imaging is necessary and whether bony or soft tissue abnormalities have to be visualized, any of these three techniques can be used.^22^ The number of available techniques, the quickly advancing technologies, and the large amount of information obtained request a high level of structured reporting to accurately and reliably describe the imaging findings.^19^ Moreover, radiologists sometimes receive a limited amount or unstructured feedback from referring colleagues, making it difficult to further improve the used terminology.^2^^,^^7^ A structured radiological report which is accepted in consensus by both musculoskeletal radiologists and orthopedic surgeons may result in the use of a clear terminology and high impact on clinical care that can be understood by all involved health professionals. With the increasing popularity of clinical prediction models that also make use of radiology reports, consensus on the contents and structure of reports becomes even more important to prevent bias in these models.^29^

The Delphi study design is a recognized method of assembling an expert panel to reach consensus on controversial topics.^8^^,^^9^^,^^27^ Randomized controlled trials that were executed by different researchers or at different times may cause heterogeneity in the data because the factors that were measured and the way in which these were measured may differ. This may limit the value of future systematic reviews because the heterogeneity may decrease the accuracy at which small differences can be observed. By gathering an expert panel of musculoskeletal radiologists and orthopedic surgeons, a general core outcome set of preferred assessments may be developed. This may limit heterogeneity and allow for higher quality evidence and serve as a core outcome set for all radiologists and especially residents, and probably ultimately decreased workload.^16^

The workload of radiologists has increased considerably over the past decades.^16^ Reaching consensus on what factors to report and what factors not to report may aid in decreasing this workload and increasing report quality.^19^ However, reaching consensus and implementing this into daily clinical work can be challenging. In order to create awareness of heterogeneous data and start the discussion about what data need to be collected through the radiology report, the aim of this study was to determine (1) which elements are considered to be important in the x-ray, MRA, and CT radiology reports for the evaluation of anterior shoulder instability, (2) the variability in which elements are considered to be important in these radiology reports, and (3) which MRI technical specifications are considered to be important.

## Materials and methods

The Delphi was systematically executed according to the recommendations of Hohmann et al, Diamond et al, and Taylor et al.^4^^,^^9^^,^^27^ The liaison (C.R.) created the online questionnaires using Castor EDC (Amsterdam, the Netherlands), communicated with the panel, and provided a summary of the previous round before starting the next round while guaranteeing anonymity of the panelists. The liaison did not take part in the questionnaires. The first round entailed the collection of elements through an open ended that were deemed relevant by at least one panelist to be mentioned in the radiographic, MRA, and CT radiology report, respectively. To prevent bias, no predetermined content was provided to the panelists. The second round rated the importance of all these factors. The third round rated the importance of these factors following an anonymous summary and measured the within-subject stability of responses.

### Recruitment of expert panelists

A panel of orthopedic surgeons and musculoskeletal radiologists was sought in order to achieve consensus across associated specialties. Specialists were eligible for recruitment in the expert panel when they had at least 3 years of experience as a shoulder specialist or musculoskeletal radiologist. Suitable candidates were recruited through the networks of the coauthors (orthopedic surgeon and musculoskeletal radiologist) and through an e-mail sent to all musculoskeletal radiologists affiliated with the Dutch Association of Radiology. There is no consensus on the number of experts needed in a heterogenous panel. Accuracy improves up to a group size of 29, so it was aimed to include at least 15 experts in each group.^27^

### First round

Baseline characteristics, the associated specialty (orthopedic surgeon or musculoskeletal radiologist), and the experience in the respective field were collected. The first round entailed the collection of elements that were deemed relevant for mentioning in the radiology report for anterior shoulder instability through open-ended questions for the three modalities, radiograph, MRA, and CT separately. Additionally, musculoskeletal radiologists were asked to mention which technical features regarding MRI they deemed to be important, such as views, MRI field strength, and sequences. Questions included: ‘*Which factors do you believe should always be mentioned in the routine x-ray report for the evaluation of patients with anterior shoulder instability?*’, the question was phrased similarly for the MRI and CT report, and ‘*Which MRI field strength, sequences, and views do you believe should always be used in the evaluation of a patient with anterior shoulder instability?*’ Following round one, all answers given by the panel were discussed with the orthopedic surgeon and musculoskeletal radiologist who were coauthors (H.W. and M.P.J.B.). Any disagreement in the descriptions of the mentioned factors was discussed by the team to formulate more appropriate wording. The resulting elements were gathered in a questionnaire using Castor EDC (Castor, Amsterdam, the Netherlands) to be used for rounds two and three.

### Round two and three

Through the e-questionnaire, the expert panel rated the elements collected during round one on a 9-point Likert scale where 1 was ‘*not important*’ and 9 was ‘*very important.*’ The questions included: ‘*How important do you believe it is to always mention the following factors in the radiology report in the x-ray evaluation of a patient with anterior shoulder instability?*’ The same was asked for the MRA and CT evaluation. The results of the second round were summarized in histograms and presented anonymously to the panel before round three. When an element reached agreement ≥80% in round two, the panel was not asked to rate these again.^27^ Consensus was defined as an element being rated between 1 and 3 (not important) or between 7 and 9 (important) by ≥ 80% and strong consensus by ≥ 90%. In round three, the panelists were asked to rate the remaining elements in a similar fashion to round two.

### Data collection and analysis

Baseline characteristics (sex, age, specialism, and experience) were presented as mean with standard deviation or median and interquartile range depending on the normality of the distribution. The Shapiro-Wilk test was used to determine the normality of the data. Consensus was presented as proportions. High variability of an element was defined as having one score between 1-3 and one score between 7-9. Data were collected using Castor EDC (Amsterdam, the Netherlands) and analyzed using Excel (Microsoft Excel 2018; Microsoft Corp., Redmond, WA, USA).

## Results

### Recruitment of expert panel and characteristics

Between May 5, 2022, and January 2, 2023, a total of 42 specialists were recruited. A total of 37 specialists responded to the first round, and 36 completed all three rounds (Table I).

**Table I:** Expert panel characteristics.

	Musculoskeletal radiologists (n = 21)	Orthopedic surgeons (n = 15)	Total (n = 36)
Age, mean (SD)	40.8 (6.0)	47 (6.0)	43.1 (6.7)
Female sex, % (n)	19.0 (16)	13.3 (2)	16.7 (6)
Years of experience as radiologist or orthopedic surgeon, median (IQR)	13 (10-17)	11 (10-18)	14 (12-20)
Years of clinical experience with shoulders, median (IQR)	10 (5-14)	19 (14-25)	10 (5-10)

*n*, number; *SD*, standard deviation; *IQR*, interquartile range.

### Round one

Following the first round, seventeen elements were identified to be mentioned in the radiology report for a radiographic evaluation, 52 elements for an MRA evaluation and 21 elements for a CT evaluation (Supplementary Table S1). Two MRA field strengths and 23 combinations of sequences and views were mentioned.

### Round two and three: radiographs

Following round two, consensus (rating of 7-9 by ≥ 80% of panelists) was reached on two elements and strong consensus (rating of 7-9 by ≥ 90% of panelists) on three elements (Table II; Supplementary Figure S1). These included the presence of a *Hill-Sachs lesion* (96%), *osseous Bankart lesion* (96%), *glenoid fracture* (96%), *greater tubercle fracture* (84%), and *loose body* (80%). Following round three, no more elements reached consensus. High variability (at least one score between 1-3 and one score between 7-9) was observed in thirteen elements (76.5%).

**Table II:** Elements that reached consensus for radiographic evaluation.

Strong consensus (≥90%)	Consensus (≥80%)
Hill-Sachs lesion presence	Greater tubercle fracture presence
Osseous Bankart lesion presence	Loose body
Glenoid fracture presence	

### Round two and three: MRA

Following round two, consensus was reached on ten elements and strong consensus on nine elements regarding the MRA report (Table III; Supplementary Figure S2). These included *which rotator cuff tendon/muscle was torn* (100%), *presence of rotator cuff tear* (100%), *presence of Hill-Sachs lesion* (100%), *presence of Bankart lesion* (96%), *presence of reverse Hill-Sachs lesion* (96%), *nature of cuff tear classified as rupture or tendinopathy* (96%), *presence of posterior Bankart lesion* (92%), *presence of osseous Bankart lesion* (92%), *rotator-cuff tear classified as partial or full-thickness tear* (92%), *presence of any type of labrum lesion* (88%), *presence of osteochondral lesion* (88%), *presence of glenoid fracture* (88%), *presence of superior labral tear from anterior to posterior* (88%), *presence of humeral avulsion of the glenohumeral ligament* (87%), *presence of bone marrow edema* (84%), *presence of glenoid bone loss* (84%), *Goutallier classification of rotator-cuff degeneration* (83%), *anatomical variants* (83%) and *presence of a greater tubercle* fracture (80%). Following round three, consensus was reached on one more element, *location of glenoid bone loss using clock-face method* (88%). High variability was observed in 45 elements (85.0%).

**Table III:** Elements that reached consensus for MRA evaluation.

Strong consensus (≥90%)	Consensus (≥80%)
Which rotator cuff tendon/muscle was torn	Presence of any type of labrum lesion
Presence of rotator cuff tear	Presence of osteochondral lesion
Presence of Hill-Sachs lesion	Location of glenoid bone loss using clock-face method
Presence of Bankart lesion	Presence of glenoid fracture
Presence of reverse Hill-Sachs lesion	Presence of SLAP tear
Nature of cuff tear classified as rupture or tendinopathy	Presence of HAGL lesion
Presence of posterior Bankart lesion	Presence of bone marrow edema
Presence of osseous Bankart lesion	Presence of glenoid bone loss
Rotator cuff tear classified as partial or full-thickness tear	Goutallier classification of rotator cuff degeneration
	Anatomical variants
	Presence of tubercle majus fracture

*MRA*, magnetic resonance imaging arthrography; *SLAP*, superior labral tear from anterior to posterior; *HAGL*, humeral avulsion of the glenohumeral ligament.

Consensus was reached on two elements regarding the technical aspects of the MRA evaluation (Supplementary Figure S2). The *coronal oblique view on T1* (81%) was considered important and the *axial view on T2 setting* (80%) was considered not important. High variability was observed in eighteen elements (85.7%).

### Round two and three: CT

Following round two, consensus was reached on four elements and strong consensus on five elements (Table IV; Supplementary Figure S3). These included the *presence of Hill-Sachs lesion* (100%), *presence of osseous Bankart lesion* (100%), *presence of glenoid fracture* (96%), *presence of glenoid bone loss* (96%), *presence of greater tubercle fracture* (92%), *presence of reverse Hill-Sachs lesion* (84%), *location of glenoid bone loss using clock-face method* (80%), *glenoid bone loss using* three-dimensional *best-fit circle method* (80%), and *glenoid bone loss using* two-dimensional *best-fit circle method* (80%). Following round three, no more elements reached consensus. High variability was observed in sixteen elements (76.2%).

**Table IV:** Elements that reached consensus for CT evaluation.

Strong consensus (≥90%)	Consensus (≥80%)
Hill-Sachs lesion presence	Reverse Hill-Sachs presence
Osseous Bankart lesion presence	Location of glenoid bone loss (clock-face method)
Glenoid fracture presence	Glenoid bone loss (3D best-fit circle method)
Glenoid bone loss presence	Glenoid bone loss (2D best-fit circle method)
Greater tubercle fracture presence	

*CT*, computed tomography; *2D*, two-dimensional; *3D*, three-dimensional.

## Discussion

The most important findings of the current study were that there was high variability among expert panelists for elements that are considered important in the radiology report of a shoulder instability patient. However, consensus was reached on five elements for the x-ray report, twenty for the MRA report, nine for the CT report, and two for the views and settings of the MRI/MRA examination.

The results should be interpreted with the following strengths and limitations in mind: Firstly, the heterogenic panel with musculoskeletal radiologists and orthopedic surgeons may provide a general impression of which elements are deemed to be important across the involved specialists. No differentiation was made between cases of first or recurrent dislocation, subluxation or dislocation, and painless or painful dislocation. The heterogenic panel may also explain the observed variability because across the two fields of expertise other elements may be considered important. Secondly, the panel only included Dutch specialists, which may not fully represent what other international specialists think is important. Thirdly, consensus for the ultrasound report was not studied. This may be important in the acute setting of anterior shoulder dislocation. Fourthly, consensus is lacking on what the best methods are to conduct a Delphi study. For example, an open discussion or more rounds can be added to increase consensus. This study followed the criteria and recommendations outlined by multiple authors to support in proper execution and reporting.^4^^,^^9^^,^^27^ The results function to start the discussion on which data researchers want to collect and how in order to decrease heterogeneity in future research. The current factors that reached consensus do not form an exhaustive list. There may be important factors that were not mentioned or did not reach consensus, such as risk factors for recurrence or that support deciding on the appropriate treatment. These may include on-/off-track Hill-Sachs lesions, distance-to-dislocation, bipolar lesions, signs of hyperlaxity, and others. Also, some factors may be less relevant in the light of anterior instability and be more important for posterior or multidirectional instability, such as the reverse Hill-Sachs lesions. This study can be a baseline for further consensus studies. One must keep in mind that, despite a standardized report, there can still be variability between readers when determining the presence of lesion or measuring their size.

The reduction of not-understood heterogeneity in what is considered important in the radiology reports should be an important aim in the near future. A structured method of reporting radiological findings and executing a radiological evaluation will improve consistency between data across studies, increase transparency of analysis, facilitate comparisons between studies, and increase the accuracy at which small differences can be found in outcomes because heterogeneity within populations may also be reduced.^6^^,^^17^^,^^19^^,^^24^ Heterogeneity between studies may be a sign of missing data, and thus a sign of introduced bias.^10^ Methods such as multiple imputation or partial deletion are commonly used to reduce the introduced bias, but these will never reach the quality of data as a homogenous database.^26^ Alongside reaching consensus on what should be measured and how, guidelines such as *the STrengthening the Reporting of OBservational studies in Epidemiology statement* also provides a guideline on how to report on missing data.^32^

Alongside the benefits of structured reporting on future research, there may also be several benefits on daily clinical practice. The reliability of healthcare within and across hospitals may be increased by consensus-based standardized reporting and the quality of future research and big data models.^21^ Framing bias, which may be introduced by providing incomplete or inadequate to the radiologist, may cause radiologists to reach a different conclusion or use different terminology.^2^ Standardized reporting may decrease the impact of framing bias, standardize terminology, and potentially make radiological terminology easier to interpret for clinicians.^5^ Also, administrative load may be decreased, which may be especially useful for residents and young colleagues and increase the satisfaction of radiologists.^15^

The importance of standardized reporting is increasing both clinically and scientifically by the rise of big data and artificial intelligence models.^3^^,^^21^ Consensus studies such as the current one that provide a structured guideline on how to report data may reduce the administrative workload. Therefore, future research should aim to increase consensus on which outcomes should be measured and how these should be measured and study the most optimal method of implementing structured reporting.^19^ Confirmation of the radiological report through surgery may support in improving the accuracy of future reports. The workload of clinicians should also be taken into account in these studies. Lastly, patient-specific factors unrelated to the radiological report may play an important role in predicting success of treatment and should also be the topic of future research.

## Conclusion

Substantial variability was observed in the scoring of important elements in the radiological for the evaluation of anterior shoulder instability, regardless of modality. Consensus was reached for five elements in the x-ray report, twenty in the MRA report, and nine in the CT report. Finally, consensus was reached on two elements regarding MRA views and settings.

## Disclaimers:

Funding: No support in the form of grants, equipment, or other items was received.

Conflicts of interest: A. van Noort is a consultant at LIMA (education and clinical research). D.F.P. van Deurzen is a paid instructor for Wright medical. W. Foppen has received research grants from NovoNordisk and Pfizer, and performed consultancy activities. The other authors, their immediate families, and any research foundation with which they are affiliated have not received any financial payments or other benefits from any commercial entity related to the subject of this article.
