# Supplementary material for: Substantial variability in what is considered important in the radiological report for anterior shoulder instability: a Delphi study with Dutch musculoskeletal radiologists and orthopedic surgeons
Source: JSES Int. 2024 Apr 8;8(4):746–50. doi: 10.1016/j.jseint.2024.03.012 (PMC11258832; doi:10.1016/j.jseint.2024.03.012)
Supplement: Supplementary Figure S1 [file mmc1.docx]

**Figure I.** Results of second and third round, x-ray elements.
