# Supplementary material for: Substantial variability in what is considered important in the radiological report for anterior shoulder instability: a Delphi study with Dutch musculoskeletal radiologists and orthopedic surgeons
Source: JSES Int. 2024 Apr 8;8(4):746–50. doi: 10.1016/j.jseint.2024.03.012 (PMC11258832; doi:10.1016/j.jseint.2024.03.012)
Supplement: Supplementary Figure S2 [file mmc2.docx]

**Figure II.** Results of second and third round, MRA elements.

PD = proton density, FS = fat-saturation, T1 = longitudinal relaxation time, T2 = transverse relaxation time, HR = high-resolution, 2D = two-dimensional, 3D = three-dimensional, ABER = abduction and external rotation, ALPSA = anterior labral periosteal sleeve avulsion, POLPSA = posterior labrocapsular periosteal sleeve avulsion, SLAP = superior labral anterior posterior, GLAD = glenolabral articular disruption, HAGL = gumeral avulsion of the glenohumeral ligament, GAGL = glenoid avulsion of the glenohumeral ligament, IGHL = inferior glenohumeral ligament, MGHL = middle glenohumeral ligament, SGHL = superior glenohumeral ligament, CHL = coracohumeral ligament, AC = acromioclavicular.
