# Supplementary material for: Substantial variability in what is considered important in the radiological report for anterior shoulder instability: a Delphi study with Dutch musculoskeletal radiologists and orthopedic surgeons
Source: JSES Int. 2024 Apr 8;8(4):746–50. doi: 10.1016/j.jseint.2024.03.012 (PMC11258832; doi:10.1016/j.jseint.2024.03.012)
Supplement: Supplementary Table S1 [file mmc4.docx]

**Table I:** All elements that were mentioned in the first round.

| X-ray | MRI | | CT |
| --- | --- | --- | --- |
| Presence of glenoid substance loss | MRI field strength of 1.5 tesla | Hill-Sachs lesion presence | Glenoid substance loss presence |
| Location of glenoid substance loss | MRI field strength of 3 tesla | Location and extension of labral lesion according to clock face method | Glenoid substance loss percentage according to best fit circle method |
| Glenoid facture presence | 3D proton density high resolution thin slice | Direction of subluxation of the glenohumeral joint | Glenoid substance loss percentage according to 3D best fit circle method |
| Osseous Bankart lesion presence | Location and extension of labral lesion in quadrants | Direction of subluxation of the glenohumeral joint | Glenoid fracture presence |
| Osseous Bankart lesion presence on old radiograph | ABER position | Glenoid shape | Osseuous Bankart lesion presence |
| Loose bony fragment presence | ABER T1 fat-saturation | Glenohumeral dysplasia | Extent of dislocation bony Bankart fragment |
| Intact sclerotic line of the glenoid | Coronal oblique T1 fat-saturation | Loose bony fragment presence | Glenoid shape |
| Shape of the glenoid | Coronal oblique T2 fat-saturation | Bone marrow edema presence | Chronic or acute glenoid bone loss |
| Greater tubercle fracture presence | Coronal oblique proton density | All labrum lesions presence | Location of glenoid bone loss |
| Hill-Sachs lesion presence | Coronal oblique proton density fat-saturation | Bankart lesion presence | Greater tubercle fracture |
| Hill-Sachs lesion involvement of bone or cartilage | Coronal oblique T1 | Posterior Bankart lesion presence | Hill-Sachs lesion presence |
| Proximal humerus fracture | Coronal oblique T2 | Perthes lesion presence | Hill-Sachs width |
| Subluxation of the glenohumeral joint | Radial proton density | ALPSA lesion presence | Hill-Sachs depth |
| Direction of subluxation of the glenohumeral joint | Sagittal proton density | POLPSA lesion presence | Hill-Sachs interval length |
| Description of glenohumeral joint space | Sagittal proton density fat-saturation | SLAP lesion presence | On- / off-track Hill-Sachs lesion |
| Light bulb sign | Sagittal T1 | Snyder classification of SLAP lesion | Reversed Hill-Sachs lesion presence |
| Hemarthrosis presence | Sagittal T1 fat-saturation | Kim lesion presence | Subluxation of the glenohumeral joint |
|  | Sagittal T2 | GLAD lesion presence | Subluxation of the glenohumeral joint |
|  | Transverse proton density | HAGL lesion presence | Glenohumeral dysplasia |
|  | Axial fat-saturation | GAGL lesion presence | Loose bony fragment presence |
|  | Transverse T1 fat-saturation | 3D multiplanar reformation |  |
|  | Transverse T2 | Osteochondral lesion presence |  |
|  | Transverse T2 fat-saturation | Labral anatomical variants |  |
|  | Transverse proton density fat-saturation | IGHL lesion presence |  |
|  | Transverse T1 | MGHL lesion presence |  |
|  | Glenoid substance loss presence | SGHL lesion presence |  |
|  | Glenoid substance loss according to best fit circle method | CHL lesion presence |  |
|  | Chronic or acute glenoid bone loss | AC ligament lesion presence |  |
|  | Glenoid fracture presence | Capsular redundancy |  |
|  | Osseous Bankart lesion presence | Rotator-cuff lesion presence |  |
|  | Location of glenoid bone loss | Involved rotator-cuff tendon |  |
|  | Extent of dislocation bony Bankart fragment | Description of rotator-cuff lesion |  |
|  | Greater tubercle fracture presence | Size of rotator-cuff tear |  |
|  | Hill-Sachs lesion presence | Description of rotator-cuff musculature |  |
|  | Hill-Sachs width | Rotator interval length |  |
|  | Hill-Sachs depth | Bicipital-labral complex status |  |
|  | Hill-Sachs interval | Paralabral cysts |  |
|  | On- / off-track Hill-Sachs lesion | Glenohumeral effusion |  |
|  | Reversed Hill-Sachs lesion presence |  |  |

3D = three-dimensional, ABER = abduction and external rotation, T1 = longitudinal relaxation time, T2 = transverse relaxation time, ALPSA = anterior labral periosteal sleeve avulsion, POLPSA = posterior labrocapsular periosteal sleeve avulsion, SLAP = superior labral anterior posterior, GLAD = glenolabral articular disruption, HAGL = gumeral avulsion of the glenohumeral ligament, GAGL = glenoid avulsion of the glenohumeral ligament, IGHL = inferior glenohumeral ligament, MGHL = middle glenohumeral ligament, SGHL = superior glenohumeral ligament, CHL = coracohumeral ligament, AC = acromioclavicular.
